# Home medication management practices and associated factors among patients with selected chronic diseases in a community pharmacy in Uganda

**DOI:** 10.1186/1472-6963-12-323

**Published:** 2012-09-18

**Authors:** Joan N Kalyango, Maurice Hall, Charles Karamagi

**Affiliations:** 1 Department of Pharmacy, Makerere University College of Health Sciences, Kampala, Uganda; 2 Clinical Epidemiology Unit, Makerere University College of Health Sciences, Kampala, Uganda; 3 Department of Pharmacy, Queens University Belfast, Belfast, UK; 4 Department of Paediatrics and Child Health, Makerere University College of Health Sciences, Kampala, Uganda

**Keywords:** Home management, Medicines management, Chronic disease

## Abstract

**Background:**

Chronic diseases are rapidly increasing and are currently the major cause of death and disability worldwide. Patients with chronic diseases experience many challenges including medicine-related problems. However, there is limited information about the home management of medicines among these patients. This study therefore was to determine home medication management practices and associated factors among patients with chronic diseases seeking care in a community pharmacy in Uganda.

**Methods:**

A cross-sectional study was conducted in a community pharmacy in Kampala from June to July 2010. A total of 207 consenting chronic disease patients or caregivers of children with chronic disease were consecutively sampled. The patients were visited at home to evaluate their drug management practices and to check their medical forms for disease types and drugs prescribed. An interviewer-administered questionnaire and an observation checklist were used to collect the data.

**Results:**

Overall home medication management was inappropriate for 70% (n = 145) of the participants (95% CI = 63.3-76.2) and was associated with perceived severity of disease (not severe OR =0.40, moderately severe OR = 0.35), duration of disease >5 years (OR = 2.15), and health worker not assessing for response to treatment (OR = 2.53). About 52% (n = 107) had inappropriate storage which was associated with inadequate information about the disease (OR = 2.39) and distance to the health facility >5 kilometres (OR = 2.82). Fifteen percent (n = 31) had no drug administration schedule and this was associated with increasing age (OR = 0.97), inadequate information about the disease (OR = 2.96), and missing last appointment for medical review (OR = 6.55). About 9% (n = 18) had actual medication duplication; 1.4% (n = 3) had expired medicines; while 18.4% (n = 38) had drug hoarding associated with increasing number of prescribers (OR = 1.34) and duration of disease (OR = 2.06). About 51% (n = 105) had multiple prescribers associated with perceiving the disease to be non severe (OR = 0.27), and having more than one chronic disease (OR = 2.37).

**Conclusions:**

Patients with chronic disease have poor home management of medicines. In order to limit the occurrence of poor outcomes of treatment or drug toxicity, health providers need to strengthen the education of patients with chronic disease on how to handle their medicines at home.

## Background

Chronic diseases are now the major cause of disability and death worldwide contributing about 60% of all deaths [1]. The burden of chronic diseases is rapidly increasing especially in developing countries where it is estimated that 79% of all deaths attributable to chronic diseases currently occur [2]. Chronic conditions are often accompanied by numerous challenges including medicines-related problems [3] because of the prolonged period over which patients take medication and the large number of drugs they usually take. The medicines-related problems include: polypharmacy, non-adherence to prescribed medicines, development of adverse events, inappropriate prescribing and dispensing, inadequate monitoring of treatment and poor home management of medicines. Most studies on medicines-related problems have looked at non-adherence, polypharmacy and inappropriate prescribing [4]. Very few studies have focussed on home management of medicines and yet this is very important for patients’ treatment outcomes. Poor home management of medicines includes poor drug storage practices, lack of medication administration schedule, use of drugs from multiple prescribers, use of discontinued medicines, expired drugs or drugs that are no longer needed and use of over-the-counter drugs which are not suitable for their condition [5].

Appropriate management of medicines is crucial among chronic disease patients. It ensures that drugs maintain their potency due to appropriate storage. It also ensures drug safety by avoiding mix-up of drugs and over dosing and reduces on wastage of resources [6,7]. Studies that have looked at home management of medicines found inappropriate practices in 10-57% of patients [5,7,8]. The factors that were associated with these practices included: gender, age, confusion between trade and generic names [9], education level [10] and number of medicines [11].

There is limited information about the home medication management practices among chronic disease patients in developing countries like Uganda and yet the conditions under which many of these patients live may encourage poor medication management practices. The limited resources in some areas may encourage patients to keep drugs that have been discontinued or those that have expired in order to use them in future. Low levels of health literacy have been found among chronic disease patients [12] and this may lead to confusion in drug names. In addition, patients may not be able to read the storage and drug use instructions or they may not be able to afford refrigerators that are required for the storage of certain drugs [13]. Furthermore the weak drug policies and regulatory bodies in some countries may not adequately control the use of drugs [14]. Patients are able to access medicines without current prescriptions implying that they can continue using discontinued medicines. This study was therefore carried out to determine home management practices and their associated factors among chronic disease patients in a community pharmacy in Kampala, Uganda.

## Methods

### Study design and setting

A cross-sectional study was conducted from June to July 2010 in a community pharmacy in Kalerwe, Kampala. Kalerwe is a peri-urban area of Kampala, the capital city of Uganda. It is both a residential and commercial area, and has a large farmers’ market. The main occupation of the people is small scale trade in foodstuffs and services. The area has a big slum and experiences frequent disease outbreaks e.g. cholera. Kalerwe is adjacent to Mulago National Referral Hospital and is served by 4 pharmacies. The community pharmacy where the study was conducted serves an average of 180 patients a day and of these about 15 patients have chronic diseases. This distribution of patients in the pharmacy reflects Uganda’s disease profile where the top ten causes of morbidity are mostly infectious diseases of short duration [15].

### Participants

Patients were included in the study if they had a chronic disease (asthma, chronic obstructive pulmonary disease, cardiovascular diseases [angina, congestive heart failure, hypertension, stroke], arthritis, thyroid diseases, diabetes mellitus, chronic hepatic disorders, chronic kidney disorders, hyperlipidemias, sickle cell disease, gastro-intestinal diseases); were on treatment for the chronic disease for at least two months; lived within one kilometre of the community pharmacy; allowed the researchers to visit their homes to observe their medication management practices; and gave informed consent to participate in the study. In the case of children, the respondent was the primary caregiver presenting at the community pharmacy during the study period. Patients with chronic diseases that did not require the patient to manage their own medicines (e.g. those where the patient went to the health facility for administration of medicines) were excluded from the study.

### Data collection

The data was collected by intern pharmacists who were trained about the study. They used a pre-tested questionnaire and an observation checklist to obtain information on participants’ socio-demographics, disease type and duration, number of prescribed drugs, frequency of administration, affordability of prescribed drugs, housing type, number of people in the household, type of health facility and distance to health facility, knowledge and perception of the disease and drugs, lifestyle characteristics, perceived adequacy of information obtained from health care provider and medication management practices including: medication duplication, storage practices, drug hoarding, sharing of medications, medication administration routine, keeping expired medicines, and having multiple prescribers. Patients’ medical forms were reviewed to confirm information about disease types and the medicines the patient was taking.

The questionnaire was translated into the main local language spoken in the area (Luganda). The patients were approached when they came to the pharmacy and informed about the study. Patients who consented to participate in the study were visited in their homes for interviews and evaluation of their drug storage practices.

#### Variable definitions

Appropriate home medication management was described as appropriate storage of medicines, having a drug administration schedule, absence of medication duplication, absence of drug hoarding or keeping expired medicines, not sharing medicines and having one prescriber for a disease condition [9].

Appropriate storage of medicines included storing of medicines in a cool dry place or refrigerator as appropriate (i.e. preferably not in bathrooms or kitchens or other humid places) and out of direct sunlight. They must have been stored so as to ensure that they could not be taken inappropriately, particularly by children. Therefore if medicines were kept on a table or low shelf or any other easily accessible place that was not locked when the household routinely had children, storage of medicines was considered inappropriate. The medicines should have been stored in their original containers as dispensed from the pharmacy (not decanted into other containers). Medicines stored in the refrigerator should have been placed in a container (e.g. plastic box) and kept separate from food and other consumables. All medicines in the service user’s home should have been stored in one place unless there were special instructions for storage. Further exceptions were made to the requirement to have medicines stored in one location for those patients that were taking medicines at times when they would be out of their homes. If such patients were storing some of their drugs at the work place, this was considered appropriate as long as the location at the work place was suitable for storage. Creams and ointments should have been stored in a separate container from other medicines.

Medication duplication was considered present if the patient had two or more medicines in the home containing the same drug or drugs of the same therapeutic class. It was classified as present if the patient indicated that they were taking the duplicated drugs concurrently or as possible if they had the medicines but were not taking them concurrently [9].

Drug hoarding was defined as keeping medicines that had been discontinued [9].

### Data analysis

Descriptive statistics were used for general description of study participants and medication management practices. Unadjusted analysis was performed with various independent variables and medication management practices using logistic regression. Odds ratios and their 95% confidence intervals were estimated. Independent variables whose p-values did not exceed 0.2 at unadjusted analysis were considered for adjusted analysis. Assessment of statistical interaction and confounding was done. Interaction was assessed using a chunk test done between full and reduced models while confounding was considered present if a variable changed the odds ratio of another by more than 10%. The data was entered in Epidata version 2.1b (The EpiData Association, Odense, Denmark) and analysis was performed in STATA 10 (Stata, College Station, TX, USA).

### Ethical issues

The study was approved by the Faculty of Medicine Research and Ethics Committee as well as the Uganda National Council of Science and Technology (Reference HS 783). All participants gave informed consent to participate in the study.

## Results

### Demographic and illness characteristics of the study participants

Of the 207 participants, 15.9% (n = 33) were caretakers of children while the rest were the patients themselves. Most of the study participants were females (66.2%, n = 137). Almost half were married (49.5%, n = 100) and the distribution among the Catholics, Anglicans and Muslims was almost similar. The most common occupation was businessperson (36.9%, n = 76) while the most common highest education level attained was secondary school (33.8%, n = 68). The most common disease was cardiovascular disease (42.5%) followed by diabetes (26.1%) (Table 1). The mean age (SD) was 49 (17.1) years. The median monthly income was an equivalent of 125 US dollars with minimum and maximum of 0 and 4000 US dollars respectively. The median number of diseases was 1 (minimum = 1, maximum = 4) while that of number of stored drugs was 4 (minimum = 0, maximum = 13). The mean number of prescribed drugs was 3 (SD = 1.2).

**Table 1:** Characteristics of 207 participants seeking care from the community pharmacy in Kalerwe, Uganda

**Variable (N)**	**Categories**	**Frequency**	**Percent**
Sex (207)	Male	70	33.8
	Female	137	66.2
Marital status (202)	Married	100	49.5
	Single	42	20.8
	Separated	17	8.4
	Widowed	43	21.3
Religion (205)	Catholic	59	28.8
	Anglican	54	26.3
	Muslim	62	30.2
	Pentecostal	25	12.2
	Other*	5	2.4
Occupation (206)	Student	11	5.3
	Housewife	22	10.7
	Businessperson	76	36.9
	Salaried employee	36	17.5
	Unemployed	50	24.3
	Other	11	5.3
Highest education level (201)	None	28	13.9
	Primary	55	27.4
	Secondary	68	33.8
	Tertiary	50	24.9
Disease (207)	Cardiovascular	88	42.5
	Diabetes	54	26.1
	Asthma	31	15.0
	Arthritis	22	10.6
	Sickle cell	18	8.7
	Others	31	15.0

### Home medication management practices

More than half of the participants had inappropriate storage of medicines (51.7%, n = 107) with 41.5% (n = 86) keeping the medicines in inappropriate locations. Some of these participants were keeping medicines on low tables or shelves that could easily have been accessed by children; while others were keeping them under the pillow; or in their pockets either as blister or strip packs or in medicine envelopes which were mostly paper and therefore could easily get torn while the medicine was in the pocket; and still others were keeping medicines out of the refrigerator when they should have been in cold storage. Although only a few patients had pill boxes, almost all the other patients improvised and stored their medicines in polyethylene bags. About 15% of the participants did not have a drug administration schedule. About 8.7% had actual medication duplication and 50.7% had multiple prescribers for their disease condition. Overall, the proportion of subjects with inappropriate home medication management practices was 70% (n = 145, 95% CI = 63.3 – 76.2%). Results of home medication management practices are summarized in Table 2.

**Table 2:** Home medication management practices of 207 participants in Kalerwe, Uganda

**Variable**	**Frequency**	**Percent**
Inappropriate storage of medicines	107	51.7
No specific location for medicines storage	13	6.3
Multiple medicines storage areas	33	15.9
Medicines not in original container	24	11.6
Multiple medicines in same container	35	16.9
Keep medicines in inappropriate room	11	5.3
Keep medicines in inappropriate location	86	41.5
No drug administration schedule	31	15.0
Have actual medication duplication	18	8.7
Presence of drug hoarding	38	18.4
Presence of expired medicines	3	1.4
Having multiple prescribers for a disease condition	105	50.7
Overall inappropriate medication management	145	70.0

Of the duplicated medicines, about 38.9% (n = 7) were analgesics, 22.2% (n = 4) were drugs for asthma treatment, 22.2% (n = 4) were for cardiovascular disease, 11.1% (n = 2) were antacids and 5.6% (n = 1) were medicines for colds.

### Factors associated with overall inappropriate home medicines management

At multivariate analysis the only factors that were significantly associated with overall inappropriate medicines management were duration of disease, perceived severity of disease and whether the health provider usually asks for response to treatment. Highest education level attained was retained in the final model because it was confounding the relationship between duration of disease and home medication management practices. Most patients with secondary school education had a longer duration of disease (> 5 years). The results of unadjusted and adjusted analysis are summarized in Table 3.

**Table 3:** Association between independent factors and overall inappropriate home medication management among 207 participants in Kalerwe, Uganda

**Variable**	**Inappropriate management n (%)**	**Unadjusted OR (95% CI)**	**P-value**	**Adjusted OR (95% CI)**	**P-value**
Education (n = 201)					
None	21 (75.0)	2.00 (0.70-5.68)	0.18	1.25 (0.40-3.93)	0.70
Primary	38 (69.1)	1.49 (0.66-3.36)	0.33	1.14 (0.48-2.74)	0.76
Secondary	52 (76.5)	2.16 (0.96-4.88)	0.06	2.30 (0.97-5.48)	0.06
Tertiary	30 (60.0)	1.00		1.00	
Disease duration (203)					
≤ 5 years	71 (63.4)	1.00		1.00	
> 5 years	70 (76.9)	1.92 (1.03-3.58)	0.04	2.15 (1.08-4.28)	0.03
Perceived severity of disease (197)					
Very severe	47 (82.5)	1.00		1.00	
Moderately severe	68 (66.0)	0.41 (0.19-0.92)	0.03	0.40 (0.17-0.94)	0.04
Not severe	21 (56.8)	0.28 (0.11-0.72)	0.008	0.35 (0.12-1.01)	0.05
Feel treatment helps (207)					
Yes	116 (67.8)	1.00			
No/Not sure	29 (80.6)	1.96 (0.81-4.76)	0.14		
Get adequate information(202)					
Yes	100 (66.7)	1.00			
No	42 (80.8)	2.10 (0.97-4.53)	0.06		
Smoke(206)					
Yes	7 (100.0)	1.00			
No	137 (68.8)	0.00	0.08		
Take alcohol(206)					
Yes	23 (60.5)	1.00			
No	121 (72.0)	1.68 (0.81-3.49)	0.17		
Kept last appointment(204)					
Yes	77 (65.2)	1.00			
No	66 (76.7)	1.76 (0.94-3.29)	0.08		
Health worker asks for response to treatment (207)					
Yes	98 (65.3)	1.00		1.00	
No	47 (82.5)	2.49 (1.17-5.34)	0.02	2.53 (1.10-5.80)	0.03

Other factors that were analyzed but which did not meet the criteria for adjusted analysis included: sex, age, religion, occupation, income, marital status, number of people and rooms in household, number of diseases and prescribed drugs, affordability of drugs, experiencing adverse effects, knowledge of disease and drugs, type of health facility and distance to health facility, time since the prescription was made and whether health worker asks patient about adverse effects, adherence or medicines affordability at review visits. The results for the unadjusted analysis of these factors have not been presented.

### Factors associated with specific inappropriate home medicines management practices

The factors associated with specific inappropriate home medicines management practices (having no medicines administration schedule, inappropriate storage of medicines, having multiple prescribers, and drug hoarding) at adjusted analysis have been presented in Table 4 and are summarized in the subsequent text.

**Table 4:** Factors associated with specific home management practices among 207 participants in Kalerwe, Uganda

**Variable**	**Adjusted OR (95**% **CI)**	**P-value**
**No medicines administration schedule**		
Age	0.97 (0.94-1.00)	0.04
Get adequate information about disease		
Yes	1.00	0.04
No	2.96 (1.06-8.26)	
Kept last appointment		
Yes	1.00	0.001
No	6.55 (2.24-19.14)	
**Inappropriate storage of medicines**		
Get adequate information about disease		
Yes	1.00	0.02
No	2.39 (1.18-4.81)	
Distance from health facility		
≤ 5 km	1.00	0.005
> 5 km	2.82 (1.36-5.83)	
Disease duration^#^		
≤ 5 years	1.00	0.06
> 5 years	1.78 (0.97-3.41)	
**Multiple prescribers**		
Number of chronic diseases		
One	1.00	0.04
More than one	2.37 (1.02-5.52)	
Perceived severity of illness		
Very severe	1.00	
Moderately severe	0.87 (0.43-1.77)	0.70
Not severe	0.27 (0.11-0.70)	0.007
Duration with disease*		
≤ 5 years	1.00	0.08
> 5 years	1.72 (0.94-3.15)	
**Drug hoarding**		
Increasing number of prescribers	1.34 (1.02-1.78)	0.04
Duration with disease		
≤ 5 years	1.00	0.05
> 5 years	2.06 (1.01-4.17)	

*# confounds the relationship between getting adequate information about the disease and inappropriate storage of medicines*.

* confounds the relationship between number of diseases and multiple prescribers.

#### Having no medicines administration schedule

Absence of a medicines administration schedule decreased with increasing age (OR = 0.97) but was higher in patients that felt they had not got adequate information about their disease (OR = 2.96), and those that did not keep their last appointment with the health provider (OR = 6.55).

#### Inappropriate storage of medicines

Inappropriate storage of medicines was associated with not getting adequate information about the disease (OR = 2.39) and longer distance to the health facility where care for the chronic disease is sought (OR = 2.82).

#### Having multiple prescribers

Having multiple prescribers was lower among patients that perceived their disease as not severe (OR = 0.27) but was higher among patients with more than one chronic disease (OR = 2.37).

#### Drug hoarding

Drug hoarding increased with increasing number of prescribers (OR = 1.35) and with duration of chronic disease of more than five years (OR = 2.06).

#### Therapeutic duplication and expired medicines

There were no factors associated with therapeutic duplication and expired medicines.

## Discussion and conclusions

Our study showed that the proportion of subjects with inappropriate home medication management practices was 70%. Furthermore, perceived severity of illness, duration of disease, and assessment for response to treatment were significantly associated with overall home medication management.

Home medicines management practices were poor with 7 in every 10 patients having inappropriate medicines management practices. The study showed that about 42% of patients did not keep their medicines in appropriate locations. However, this estimate is likely to have been an under-estimate since some patients may have reported what they thought was right and not necessarily what they do. This problem was minimized by observations of storage practices where patients allowed us to do so. In some cases, the patients could not afford refrigerators yet their drugs required cold storage. The findings of our study are similar to what was found in a study done in Sudan in which appropriate storage was found in 51.2% of the households [10]. Poor medicines storage practices could lead to deterioration of medicines, loss of potency, development of resistance and even degradation of medicines into toxic products.

Actual medication duplication was found in 8.7% of the patients. The proportion of patients with actual medication duplication in our study was lower than that reported in a study done in Australia (20%)[5], and in a study in California among elderly patients where 24.2% had unnecessary therapeutic duplication [11]. The populations in those studies were different from our study population. The study in Australia had patients with high risk of medication problems while that in California was in elderly patients who also have a high risk for medication problems. The most commonly duplicated drugs in our study were analgesics in contrast to the study in Australia where cardiovascular drugs were the most commonly duplicated. Although medication duplication was not related to age, education level or multiple prescribers in our study, it is possible that some patients were overwhelmed by the various brands of their medicines available on the market. This high medication duplication is likely to increase the risk of toxicity among patients as well as wastage of resources.

Our study showed that 15% of patients did not have a medication administration schedule. This was lower than what was found in a study in Australia where about 30% of respondents did not have a medication administration schedule [5]. Patients with no medication administration schedules may be non-adherent to their regimens, thus increasing the risk of non-response to treatment regimens. In addition, they may get toxic reactions to medicines if they take them at times that are very close to each other. Patients with no medicines administration schedules may also be generally non-adherent to other non-drug treatment requirements. This is supported by findings in our study of an association between having missed the last appointment for medical review with the health provider and lack of medicines administration schedule.

Only 1.5% of respondents had expired medicines, a figure that was much lower than what was reported in a previous study in Australia (20%) [5]. The very low figure of expired medicines in our study may have been an underestimate. We found that in some instances the medicines had been removed from their original containers at the pharmacy and were packed in envelopes which did not indicate the expiry date. In such cases we were not able to determine whether the drugs were expired or not. Patients who ingest expired medicines may fail to obtain adequate response from administered medicines and may also get toxic reactions.

Multiple prescribers were common in our study with about half of the respondents (51%) having more than one prescriber for the same medical condition. However, this may be an overestimate because patients with more than one chronic disease especially if it was not in the same specialty may have been visiting different prescribers for the different chronic conditions. We tried to minimize this problem by specifically inquiring if the patient got prescriptions from these different prescribers for the same condition. Patients that did not know their disease well may not have been able to distinguish between the disease conditions managed by different prescribers. Our findings are similar to what was reported in Australia where 49% of patients had medicines prescribed by more than one doctor [5]. Multiple prescribers for the same disease condition may lead to therapeutic duplication and drug interactions in cases where the different prescribers may not be aware of what other medicines the patients are taking. Strategies to integrate the care of patients with multiple chronic conditions should also be devised so as to avoid the problems that arise from patients seeking care from different prescribers.

### Factors associated with inappropriate home medication management practices

Patients who perceived their disease as moderately severe had 60% reduction in inappropriate medicines management compared to those who perceived their disease as very severe. In addition, patients who perceived their disease as not severe had 65% reduction in inappropriate medicines management compared to those that perceived their disease to be very severe. Our findings are similar to those in the study done in Australia where severity of illness was associated with medication management practices [9]. Patients who perceive their disease as very severe may take more than the recommended dosage, or take multiple medicines or visit more health providers, all of which are likely to lead to inappropriate medication management practices. Indeed in this study we found that patients that perceived their disease as not severe were less likely to have multiple prescribers for their disease condition. The multiple prescribers further compounded the problem of home management of medicines increasing drug hoarding by 35% with increasing number of prescribers.

Longer duration of disease was associated with inappropriate medication management practices. Patients who had disease for more than 5 years were twice as likely to have inappropriate medication management practices. Patients with prolonged disease may accumulate drugs over time and misuse them, and they may be more likely to visit multiple prescribers in search of a cure. In addition, they may perceive their disease as very severe and may take more than the recommended dosage, or take multiple medicines or visit more health providers in an effort to obtain relief, all of which are likely to lead to inappropriate medication management practices. In this study, there was higher drug hoarding among patients with duration of disease of more than five years. In addition, patients that had longer duration of disease had higher number of chronic diseases which in turn was associated with having multiple prescribers.

Our study showed that poor communication and guidance by the health workers was associated with poor medicine management practices. Patients who reported that the health workers did not ask them about their response to treatment were 2.5 times more likely to have inappropriate medication management practices. This may be indicative of inadequate general management of the patient’s condition due to poor communication and guidance by the health worker. In addition, patients that felt that they had not received adequate information about their disease condition were more likely not to have a medicines administration schedule and had inappropriate medicines storage practices. This calls for better communication between the various health providers (physicians, nurses, and pharmacists) and the patients.

Although highest level of education was not statistically significant, it had a confounding relationship with duration of disease. A bigger proportion of patients with secondary school education had longer duration of disease than other education categories and that was probably the reason why it appeared as an important variable to consider since duration of disease was retained as an important variable.

Our study did not show significant association between overall medication management practices and socio-demographic characteristics, disease characteristics of patients, drug related factors, home environment, type of facility the patient usually attends, and lifestyle characteristics. Our findings are similar to those of Hutchison and others [16] in USA who did not report any association between adverse events and medication management practices. However, our findings contrast with a study done in Palestinian households which reported that family size, age, and education level were associated with the storage of antibacterial agents [17]. These differences may be due to the fact that the study in Palestinian households focussed on antibacterial agents whereas our study involved a wide range of drugs used in chronic diseases. Our findings also contrast with the study by Sorensen and others in Australia [5] which reported that the number of medications was associated with poor medication management practices.

The lack of association between medication management practices and many of the factors in our study suggests that these factors were not important predictors of medication management practices in the population that we studied. Secondly, we may not have had sufficient power to detect associations where they existed for some of the variables.

In our study, some socio-demographic characteristics were associated with specific medicines management practices. There was a reduction in lack of medicines administration schedules in older chronic disease patients and longer distance to the health facility was associated with inappropriate storage of medicines. Older patients may have had the chronic disease longer and have therefore learnt how to effectively manage it by having proper medicines administration schedules or they may try to organize routines in the way they administer their medicines in order to overcome the problems of forgetfulness that increase with age.

Our study was limited in that in many instances, we were not able to enter patients’ houses and evaluate their medicines storage practices. It is therefore likely that patients may have underestimated their poor medicines storage practices in cases where it was obvious what was expected e.g. refrigeration of insulin.

We may not have had adequate power to detect associations for some of the variables that have been related to medicines management in the literature. This is more so because we had to combine several variables to come up with the outcome variable of overall appropriate medicines management practices. The small proportion of respondents with appropriate medicines management practices resulted in small numbers in some categories which may have affected the power of our study. In addition, we had some missing information on some variables further reducing the effective sample size. Associations with expired medicines could not be determined because of the small number of persons that had expired medicines.

The variable “appropriate medicines management” was made up of several items which were equally weighted in the combination and yet some of the items may have had stronger implications for inappropriate medicines management.

A major strength of our study was it was population based since patient’s medicines were evaluated in their homes. Even where we could not view where patients kept their medicines, they could bring them out and evaluate what they had. This enabled us to get valid information about the medicines kept at home rather than relying on patients recall on what was in their medicines storage areas.

In conclusion, home medication management practices among the chronic disease patients were poor. Patients that perceived their disease to be very severe, those with longer duration of disease, and those who the health worker did not assess for response to treatment were more likely to have inadequate medication management practices. Health workers need to strengthen the patients’ education about their medicines especially about the storage and other aspects of home medication management. In addition, the health workers should monitor chronic disease patients more closely. There is need for stronger drug regulation so that patients do not get medicines without proper and current prescriptions.

## Competing interests

The authors declare that they have no competing interests.

## Authors’ contributions

JNK conceived the study. JNK, MH and CK participated in the design of the study. JNK participated in the implementation of the study, and data analysis. JNK, MH and CK participated in the interpretation of results. JNK made the first draft of the manuscript. JNK, MH, and CK revised the draft manuscript and they all read and approved the final manuscript.

## Pre-publication history

The pre-publication history for this paper can be accessed here:

http://www.biomedcentral.com/1472-6963/12/323/prepub
